# O7-4 Temporal trends in sport participation according to WHO physical activity guidelines and its effect on weight status: a French school-based study between 2015 and 2019

**DOI:** 10.1093/eurpub/ckac094.052

**Published:** 2022-08-29

**Authors:** Maxime Luiggi, Olivier Rey

**Affiliations:** ISM, UMR CNRS 7287, Aix-Marseille Université, Marseille, France

**Keywords:** weight status, obesity, sport, physical activity, social inequalities

## Abstract

**Background:**

Insufficient physical activity (PA) is an important risk factor for overweight and obesity among adolescents, both causes of cardiovascular diseases. Sport is the largest contributor to achieving the World Health Organization (WHO) PA levels guidelines. However, few studies have investigated temporal trends in weekly sport participation in relation to adolescents' weight status. The main objective of this study was to compare sport participation to weight status and investigate their relationship over time.

**Methods:**

Two data collections were conducted during spring 2015 (n = 1019) and 2019 (n = 1112) in 30 French high schools. Adolescents reported their age, sex, height, weight, parents' socioeconomic status (SES) and hours of sport per week. Body mass index was calculated and classified into three categories (normal weight, overweight, obese) according to the International Obesity Task Force. Three sports groups were created according to WHO PA guidelines: no sport (NS), sport less than 7 hours a week (7-) and sport more than or equal to 7 hours a week (7+). Binary logistic regressions adjusted for age, sex and SES were performed to evaluate sport participation, weight status and their relationship over time.

**Results:**

Between 2015 and 2019, prevalence of overweight and obesity increased from 9.1% to 14.1%, with this increase greater among low-SES girls. We observed an increase in both the proportion of adolescents playing 7+ hours of sport (8.0% to 12.3%) and those not playing sport (34.2% to 35.9%). These changes varied according to adolescents' SES. In 2019, low-SES adolescents were 1.5 times less likely to play sport (95% CI:1.17-1.99) while no change was observed among high-SES adolescents. Finally, 7+ adolescents were no more likely to be overweight or obese in 2019 compared to 2015. Contrarily, NS and 7- adolescents were 1.7 and 1.8 times more likely respectively to be overweight or obese in 2019 compared to 2015.

**Conclusion:**

These results confirm the adequacy of WHO PA recommendations to counter the rise of overweight and obesity, and show that enough hours of sport participation alone could help stabilize overweight and obesity prevalence, without controlling for other forms of PA.

